# Evaluation of computed tomography obstruction index in guiding therapeutic decisions and monitoring percutanous catheter fragmentation in massive pulmonary embolism

**DOI:** 10.1016/S1674-8301(11)60057-2

**Published:** 2011-11

**Authors:** Tongfu Yu, Mei Yuan, Qingbo Zhang, Haibing Shi, Dehang Wang

**Affiliations:** Department of Radiology, the First Affiliated Hospital of Nanjing Medical University, Nanjing, Jiangsu 210029, China.

**Keywords:** pulmonary embolism, CT angiography, scoring system, catheter fragmentation

## Abstract

In the present study, we evaluated computed tomography pulmonary angiography (CTPA) in guiding therapeutic decisions and monitoring patients undergoing percutaneous catheter fragmentation in acute massive pulmonary embolism. From Jan 2003 to Dec 2009, 35 patients were diagnosed with acute massive pulmonary embolism by CTPA (T0) and treated with percutaneous catheter fragmentation. The severity was assessed by CT obstruction index (Qanadli index) and compared with Miller index. CTPA, oxygen saturation (SaO_2_) and pulmonary artery pressure were performed as follow-up index. The mean percentage of Qanadli index was (55±13)% (range, 40%-75%), and Miller index was (62±15)% (range, 45%–85%). Correlations between them were statistically significant (*r* = 0.867, *P* < 0.0001). The Qanadli index showed significant reduction [T0: (55±13)%; T1: (12±10)%; *P* < 0.001] in 33 patients. Significant correlation was observed between the Qanadli index, SaO_2_ (*r* = 0.934), and pulmonary artery pressure (*r* = 0.813). The Qanadli index provides an accurate method for distinguishing massive pulmonary embolism from sub-massive pulmonary embolism. It can be used to determine therapeutic options and monitor clinical outcomes.

## INTRODUCTION

Computed tomography pulmonary angiography (CTPA) has become the first-line technique for the detection of emboli in pulmonary arteries[1]–[4], especially with the introduction of multi-detector CT (MDCT) techniques. It has been routinely used as an accurate method to diagnose pulmonary embolism (PE) in many institutions. In addition to demonstrating the presence of pulmonary emboli, a few studies[5]–[7] have designed several indexes to quantify the severity of obstruction on CTPA. To our knowledge, the obstruction index of Quanadli *et al*.[6] provides a relatively convenient tool to quantify the degree of vascular obstruction with a high accuracy. However, the usefulness of Qanadli index for stratifying the risk of patients especially in distinguishing massive PE from sub-massive PE, determining therapeutic decisions and monitoring clinical outcomes in follow-up studies of PE remains underinvestigated.

Patients with massive PE defined by fatal hemodynamic consequences have mortality up to more than 30%[8]. Its potentially fatal consequence makes it necessary to distinguish it from sub-massive PE. Moreover, the management of massive PE is significantly different from that fro sub-massive PE. The standard medical treatment of PE is systemic intravenous thrombolysis[14]. Modern catheter-directed therapy (CDT), which is defined by the fundamental principles of mechanical clot fragmentation, clot aspiration, and intraclot thrombolytic injection by using existing low-profile catheter systems (<10 F), is a valuable addition in the management of massive PE[15]. It is selected in highly compromised patients with too high bleeding risk for thrombolysis or insufficient time for systemic thrombolysis to be effective[16].

CDT is a relatively safe and highly effective treatment for massive PE, and several studies have evaluated the safety and effectiveness of modern CDT as a potential alternative or early treatment option in patients with massive PE[15],[17],[18]. Nevertheless, the outcomes for individuals undergoing modern CDT have been under-investigated, especially with MDCT for follow-up evaluation. The primary objective of our study was not only to investigate whether the CT obstruction of Qanadli index can be used to stratify vascular obstruction and to guide the therapeutic decisions but also to monitor efficiency and clinical outcomes in patients treated with CDT during hospital stay.

In our series, all the patients undergoing CDT were treated with catheter intraembolus fragmentation, which is the most common technique performed among CDT that of combined with or without catheter directed lysis[19],[20].

## MATERIALS AND METHODS

### Patients

From January 2003 to December 2009, 165 consecutive patients without underlying cardiopulmonary disease were enrolled in the study. Of all the patients, 93 were men (56.4%) and 72 women (43.6%), with a mean age of 64 years (range, 22-87 years). These patients had a high clinical suspicion of PE according to the Wells score[21],[22] and were referred clinically for CTPA within the 24 h after admission. All the patients were scanned on a 16-MDCT scanner (Siemens emotion 16-detector CT, German). One hundred and four of 165 patients were diagnosed as PE by MDCT at the time of initial diagnosis (T0). The percentage of vascular obstruction was calculated according to the Qanadli index[6]. Other diagnostic tests included O_2_ saturation (SaO_2_) and pulmonary artery pressure (PAP) (*n* = 156). A follow-up evaluation based on the same protocol was proposed after the initial event (T1) with the mean interval time (45±6) d.

A pulmonary arterial obstruction of 40% or greater on CT scan identifies more than 90% of patients with hemodynamic impairment[6]. The inclusion criteria for our study was obstruction of 40% or greater on CT in patients without underlying cardiopulmonary disease. Forty patients were excluded from the initial population because of a diagnosis of sub-massive PE. Fourteen additional patients were excluded for the other following reasons: patients did not undergo follow-up evaluation[11]; the image quality of CT angiogram is technically suboptimal at T0 or T1[3].

Thirty-five of the remaining 50 patients underwent percutaneous catheter fragmentation, 21 were men (60%), and 14 women (43.6%), with a mean age of 54 years (range, 22-75 years). Fourteen of the 35 patients underwent percutaneous catheter fragmentation with local thrombolysis after the failure of systemic thrombolysis. The failure of systemic thrombolysis was based on several comprehensive factors according to the clinicians' evaluation, including symptoms (presence of dyspnea), clinical index (PAP did not decrease, and SaO_2_ did not improve) and all the patients underwent CT scanning and the Qanadli index did not decrease obviously. Twenty-one patients were performed with percutaneous catheter fragmentation as a first-line treatment with or without local thrombolysis. Two patients did not benefit from CDT and were sent for surgical thrombectomy.

### CT protocol

All CTPA examinations were acquired with 16-detector-row CT (Siemens emotion 16-detector CT, German) using 16×1.2 mm collimation, 110 kV, 300 mA, a pitch of 1.0, and 0.6 sec rotation. All transverse images were reconstructed with 1.5-mm slice thickness and 0.5-mm overlap with a matrix of 512×512 pixels. All CT images were acquired in a caudo-cranial direction from the level of the diaphragm to the lung apices in the mean duration of 5-7 sec for data acquisition. Patients received a total dose of 70-90 mL of 300 mg/mL Iohexol (Omnipaque 300; Amersham Health Ltd. Shanghai, China) according to the body weight of patients at a rate of 4 ml/s. A bolus tracking method was applied with the region of interest (ROI) in the pulmonary trunk. The trigger threshold was set at 100 HU and a start delay of 6 sec after reaching the trigger threshold was used. Original reports had been based on the evaluation of thin axial images. Multiplanar reconstructions (MPR) and maximum intensity projections (MIP) were used at the discretion of the interpreting radiologist.

Images were reviewed on an independent workstation (Siemens emotion Systems) by two radiologists experienced in CTPA imaging (> 5 y of experience) at standard mediastinal windows (center, 50 H; level, 350 H). Both observers were blinded to the patient's clinical data. The observers were free to review MPR and MIP and to change the window and level settings. The diagnostic criterion of embolism was the presence of endoluminal clots on CT scans. Central emboli included emboli within the main arteries, lobar arteries, or both. Peripheral emboli consisted of endoluminal clots within segmental and/or subsegmental branches. Each investigator was asked to score vascular obstruction. The percentage of vascular obstruction was calculated according to the formula described by Qanadli *et al*.[6] as follows:

The percentage of obstruction=Σ(*n.d*)/40×100

where *n* = the number of segmental branches arising distally (minimum, 1; maximum, 20) and *d* = degree of obstruction (minimum, 0; maximum, 2). The arterial tree of each lung was assigned as 10 segmental arteries (three to the upper lobes, two to the middle lobe and to the lingula, and five to the lower lobes). Values for *n* ranged from a minimum of 1 (one segment obstructed) to a maximum of 20 (obstruction of both the right and left pulmonary arteries). Values for *d* were assigned to provide information about the perfusion distal to the embolus. The maximum CT obstruction index was (20 segments×2), and the total Σ(*n*×*d*) product was divided by 40 and multiplied by 100 according to the formula {[Σ(*n*×*d*) / 40]×100}.

All patients underwent enhanced CT scan at the time of initial diagnosis (T0) and after the initial event (T1) with the mean interval time (45±6) d as follow-up based on the same protocol.

### Selective pulmonary angiography protocols and percutaneous fragmentation and thrombolysis

**Fig 1:**
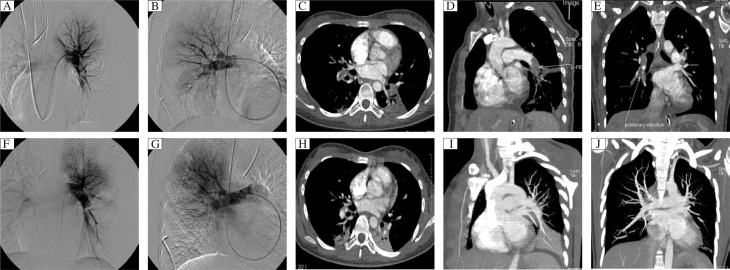
14- year-old female with dyspnea for 4 d and hypotension for 1 d. A and B: Supraselective angiogram shows multiple emboli. First investigator scored arterial obstruction as 16 and perfusion as 7; Miller index was 67%. Second investigator scored obstruction and perfusion as 16 and 13, respectively; Miller index was 85%. C-E: Axial transverse and reconstruction CT scan shows occlusive clots in right middle lobar artery and bilateral inferior lobar pulmonary artery (arrow). First investigator calculated Qanadli index as 50%; second investigator scored as 50%. F and G: After catheter fragmentation, supra-selective angiogram was performed in the real time of therapy; the restoration of blood flow in left pulmonary artery improved obviously; however, in right pulmonary vasculature the blood flow did not improved immediately. H-J: Fifteen days after therapy, the patient did CTPA as a follow-up evaluation. The endoluminal filling defects in bilateral lung almost disappeared. Doctor A scored Qanadli index as 5%; Doctor B scored as 2.5%.

After measurement of the PAP, pulmonary angiography with a digital subtraction system (Siemens AXIDM ARTIS FA) was performed to identify the embolus in all patients who would be treated with mechanical fragmentation. The pulmonary arterial obstruction was scored using the angiographic index described by Miller *et al*. (Miller index)[23]. Then, the pigtail catheter was inserted to the embolus site and the emboli were fragmented under the rotation of pigtail catheter (***Fig. 1***). Urokinase (Lizhu, Zhuhai, China) at a dose of 100,000 units was then administered through the catheter. Fragmentation and thrombolysis were repeated till symptoms and SaO_2_ improved. The total dose of urokinase was no more than 300,000 units. Finally, pulmonary angiography and PAP were performed again to monitor the results of the therapy. After the procedure, additional systemic urokinase (200,000-400,000 units, twice a day) was administered intravenously for 3 to 5 d. During thrombolysis, low molecular weight heparin (LMWH, 4000 nits, bid) was simultaneously administered. Oral warfarin was started one day before heparin was stopped, which would last for at least 6 months.

The Miller index was scored by two other investigators who were blind to the CT findings, which is defined as follows:

Miller index = Σ (*n.d*) / 34×100

where *n* = the number of segmental branches arising distally (minimum, 1; maximum, 16) and *d* = the degree of obstruction which divides each lung into three zones and uses a four-point scale (minimum, 0; maximum,3). In the right pulmonary artery, the number of segmental branches is assigned nine (three to the upper lobe, two to the middle lobe, and four to the lower lobe), whereas in the left pulmonary artery there are only seven segmental arteries (two to the upper lobe, two to the lingula, and three to the lower lobe). The presence of segmental emboli, regardless of the degree of obstruction, is scored 1 point. Proximal emboli are scored a value equal to the number of segmental arteries arising distally. The maximal score of obstruction is 16. An additional factor was assigned to provide the residual perfusion distal to the embolus. Each lung is divided into upper, middle, and lower zones and reduction of peripheral perfusion is scored by using a four-point scale: 0 points, normal perfusion; 1 point, moderately reduced perfusion; 2 points, severely reduced perfusion; 3 points, perfusion is absent. The maximal score of reduced perfusion is 18. Thus, the maximal Miller index is 34 per patient.

### Statistical analysis

Data were expressed as mean±SD and median. In the interventional therapeutic group, inter-observer variability was determined for the date of both Qanadli indexes and Miller indexes and subsequently analyzed by linear regression equations. Spearman's rank correlation coefficients were used to assess the correlation between the Qanadli index and SaO_2_ and PAP in pre- and post-therapy. *P* value < 0.5 was considered statistically significant. All computations were performed with SPSS 15.0.

## RESULTS

The final study group included thirty-five patients, including 21 men (60%), and 14 women (43.6%), with a mean age of 54 years (range, 22-75 years). The mean percentage of vascular obstruction by the index of Qanadli *et al*. was (55±13)% (range, 40%-70%). There was a high correlation coefficient between scores obtained by both investigators (*r* = 0.924, *P* < 0.01, ***Table 1***). The mean percentage of vascular obstruction by the Miller index was (62±15)% (range,45%–85%). A high correlation coefficient was observed by both investigators (*r* = 0.902, *p* < 0.01). Correlations between the obstruction CT index and Miller index were found to be statistically significant (*r* = 0.867, *P* < 0.01, ***Table 2***).

**Table 1:** The Qanadli index before (T0a) and after (T1a) treatment

Index	CT index 1	CT index 2	Mean value of CT index^a^
T0a	56±15(range,40-72)	52±10(range,40-68)	55 ±13(range,40-70)
T1a	14±13(range,0-27)	11±8(range,0-22)	12±10(range,0-25)

^a^Calculated as the mean of percentage obtained by the two investigators.

(%)

**Table 2:** Correlation coefficients between the Qanadli index and Miller index in T0

Index	Mean value of CT index	Mean value of miller	Correlation coefficients
T0	(55 ±13)%(range,40%-70%)	(62±15)%(range,45%-85%)	*r* = 0.867

^a^Calculated as the mean of percentage obtained by the two investigators.

Thirty-three of 35 patients benefited from the technical success of catheterization and fragmentation.Two cases did not show improvement in blood flow and died during the surgical embolectomy attempt.

**Fig. 2:**
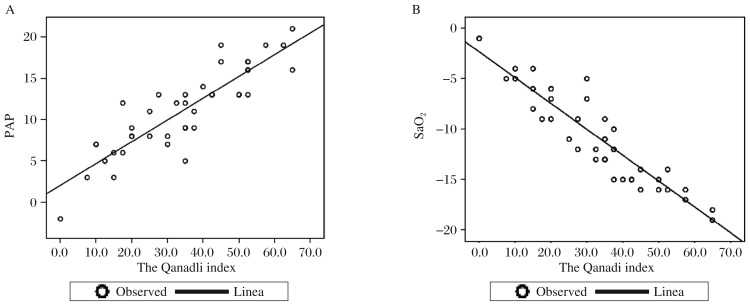
the correlation analysis between the Qanadli index and PAP and SaO_2_. A: correlation between the Qanadli index and PAP (*r* = 0.831). B: correlation between the Qanadli index and SaO_2_ (*r* = 0.934). PAP: pulmonary artery pressure.

After the interventional procedure (T1), SaO_2_ in the 33 patients increased from (86.2±4.5)% to (96.1±3.2)% (*P* < 0.001). The post-procedure mean PAP decreased from (34.2±4.8) mmHg to (25.2±5.1) mmHg (*P* < 0.001). At T1 time, CTPA was performed again, and the Qanadli index was assessed by the same investigators who scored the CT index before management. The mean Qanadli index decreased to (12±10)% (range, 0%-25%) with a high correlation coefficient between scores obtained by both investigators (*r* = 0.914, *P* < 0.0001, ***Table 1***, ***Fig. 1***). There was a significantly statistical significance between the Qanadli index and SaO_2_ (*r* = 0.934), and PAP (*r* = 0.813) in pre- and post-therapy (***Fig. 2***).

There were no major complications during the treatment in 34 patients. Intracranial hemorrhage occurred 3 days after thrombolysis in one patient who had a history of atrial fibrillation and cerebral infarction. Fortunately, the intracranial bleeding site was just the obsolete cerebral infarction area. His condition was improved later.

## DISCUSSION

The appropriate therapeutic strategy for patients with PE requires accurate risk stratifications by rapidly assessing available clinical parameters. The three key components for risk stratification are: 1) clinical evaluation, 2) determination of cardiac biomarker levels such as troponin; 3) estimation of the right ventricular size and/or function by CT scan and/or echocardiography[16].

Because of the potentially fatal hemodynamic consequences of massive PE, the therapeutic plan should be fundamentally distinct from that for deep venous thrombosis or sub-massive PE. Right ventricular enlargement is a suitable and the earliest sign of poor prognosis and determines the right ventricular function (RVD). A CT obstruction of 40% or greater will identify more than 90% of patients with right ventricular dilatation, especially in patients with normal cardiopulmonary reserve. Alternately, a CT obstruction index of less than 40% would be unlikely in the presence of a PE with acute right ventricular dysfunction[6],[24]. With the development of MDCT, emboli located at each level of the lung (from the main pulmonary artery to the sub-segmental level) can be coded on CT angiograms, allowing precise assessment of the percentage of obstruction. Thus, it needs an objective and reproducible tool to quantify obstruction seen on helical CTA. A few studies have designed several indexes with MDCT to precisely and objectively quantify pulmonary arterial tree obstructions. A new CT index defined by Qanadli *et al*.[6] provided a convenient, objective and reproducible tool to quantify obstruction seen on helical CTA. Another scoring system designed by Mastora *et al*. [25] provided a more precise calculation system of the percentage of vascular obstruction. Besides a higher degree of complexity, the accuracy of this system has not been validated. A 5-point scale for determination of arterial surface obstruction used by CT angiographic score of Mastora *et al*.[25] makes the estimation more subjective when comparing with the CT index of Qanadli *et al*.,[6] which attributes the same value to both the filling defects and perfusion obstruction.

The accuracy of the CT index by Qanadli *et al*.[6] has been evaluated in a few published articles[6],[7]. Moreover, it can be used as an objective and reproducible tool to stratify the risk of patients, especially playing a role in distincting massive PE from submassive PE. In massive PE, the accuracy of the Qanadli index has not been investigated. In our series, after calculating the CT index by Qanadli *et al*.,[6] the obstruction of 40% or greater was considered massive PE in patients without underlying cardiopulmonary disease. Correlations between the Qanadli index and Miller index were found to be statistically significant.

The usefulness of helical CT for guiding therapeutic decisions and monitoring therapeutic outcomes in follow-up studies of PE has not been fully investigated. Modern CDT was selected in highly compromised patients with too high bleeding risk for thrombolysis or insufficient time for systemic thrombolysis to be effective. There are several reasons for evaluating and monitoring the CT index in patients who received pigtail catheter fragmentation. Firstly, catheter embolectomy involves both a diagnostic and therapeutic component, so both CT and selective pulmonary angiography could be performed and compared in patients with severe pulmonary embolism. In our series, correlations between the obstruction CT index and Miller index were found to be statistically significant. The finding is in agreement with previous reports, which underlie high correlation between the CT index with Miller index[6]. Secondly, despite the lack of an official treatment indication, experts have recommended the integration of CDT into a potentially life-saving treatment algorithm. A recently published systematic review and meta-analysis by William *et al*.[15] suggested that at experienced centers, CDT should be considered as a first-line treatment for patients with massive PE. Among CDT, the rotating pigtail fragmentation, which is safe, cheap and widely available, is the most common performed technique. However, the outcomes for individuals undergoing pigtail fragmentation have been under-investigated, especially with MDCT for follow-up evaluation. In our research, during fragmentation and thrombolysis, the SaO_2_ and PAP increased significantly. In real time procedure, the mean percentage of Miller index decreased from (62±15)% (range, 45%-85%) to (22±10)% (range,10%-35%). Because of catheter fragmentation, the occlusive central embolus is dispersed to the peripheral arteries, which may in some instances resulted in distal embolization. Although adjunctive local thrombolysis would be used to accelerate the efficacy of local pharmacological and spontaneous lysis, the clinical outcomes cannot have been monitored in real time by means of angiography. By MDCT, which allows a noninvasive depiction of endoluminal clot and good visualization of the peripheral pulmonary arteries, the CT obstruction index can be quantitatively acquired to monitor therapeutic outcomes in follow-up studies. Between T0 and T1, we observed a significant reduction in the mean percentage of obstruction of the pulmonary arterial bed on CT angiograms (T0: (55±13)%; T1: (12±10)%; *P* < 0.001). Two cases did not benefit from the catheter therapy because of severe hemodynamic compromise, and died after trial of surgical thrombectomy (mortality 5.71%). We found a complete resolution of endoluminal clots at 6-week follow-up in 18 patients (51.4%), a little higher percentage than those previously reported on CT angiography follow-up studies[25]-[27]. This variation may be explained by the differences in therapeutic options as well as the severity of percentage of obstruction. During clinical follow-up (range, 1–5 years), no patients had recurrence of PE.

There were no major complications related to the catheter fragmentation procedure in our study. However, intracranial bleeding occurred in one patient who had a history of atrial fibrillation and cerebral infarction, though the total dose of the thrombolysis drug is relatively low compared with standard therapy. We acknowledge several limitations of the present study. Firstly, our results are limited by its small number of patients. Secondly, the statistical significance of clinical outcomes between pigtail catheter fragmentation and systemic intravenous thrombolysis has not been fully investigated. In our series, the rate of resolution was a little inferior than previous reports. However, variation exists for comparison with both therapeutic modalities i.e. degree of obstruction, and interval time for follow-up evaluation. More experience is needed to estimate and compare the therapeutic outcomes between them.

In conclusion, the CT obstruction index used in this study enables quantitative assessment of acute PE severity on spiral CT angiograms. The percentage of obstruction showed a strong correlation with the sign of RVD from echocardiography or chest CT scan in patients with acute PE and no underlying cardiopulmonary disease. The CT obstruction index could be used to guide therapeutic decisions and to monitor patients requiring an objective repetitive evaluation.
